# Measurement of Heat Dissipation and Thermal-Stability of Power Modules on DBC Substrates with Various Ceramics by SiC Micro-Heater Chip System and Ag Sinter Joining

**DOI:** 10.3390/mi10110745

**Published:** 2019-10-31

**Authors:** Dongjin Kim, Yasuyuki Yamamoto, Shijo Nagao, Naoki Wakasugi, Chuantong Chen, Katsuaki Suganuma

**Affiliations:** 1 Department of Adaptive Machine Systems, Graduate School of Engineering, Osaka University, Osaka 565-0871, Japan; djkim@eco.sanken.osaka-u.ac.jp; 2 The Institute of Scientific and Industrial Research, Osaka University, Osaka 567-0047, Japan; suganuma@sanken.osaka-u.ac.jp; 3 Specialty products development, Tokuyama Co., Yamaguchi 746-0006, Japan; yasu-yamamoto@tokuyama.co.jp; 4 Division of system development, Yamato Scientific Co., Ltd., Tokyo 135-0047, Japan; naoki.wakasugi@yamato-net.co.jp

**Keywords:** power cycle test, SiC micro-heater chip, direct bonded copper (DBC) substrate, Ag sinter paste, wide band-gap (WBG), thermal resistance

## Abstract

This study introduced the SiC micro-heater chip as a novel thermal evaluation device for next-generation power modules and to evaluate the heat resistant performance of direct bonded copper (DBC) substrate with aluminum nitride (AlN-DBC), aluminum oxide (DBC-Al_2_O_3_) and silicon nitride (Si_3_N_4_-DBC) ceramics middle layer. The SiC micro-heater chips were structurally sound bonded on the two types of DBC substrates by Ag sinter paste and Au wire was used to interconnect the SiC and DBC substrate. The SiC micro-heater chip power modules were fixed on a water-cooling plate by a thermal interface material (TIM), a steady-state thermal resistance measurement and a power cycling test were successfully conducted. As a result, the thermal resistance of the SiC micro-heater chip power modules on the DBC-Al_2_O_3_ substrate at power over 200 W was about twice higher than DBC-Si_3_N_4_ and also higher than DBC-AlN. In addition, during the power cycle test, DBC-Al_2_O_3_ was stopped after 1000 cycles due to Pt heater pattern line was partially broken induced by the excessive rise in thermal resistance, but DBC-Si_3_N_4_ and DBC-AlN specimens were subjected to more than 20,000 cycles and not noticeable physical failure was found in both of the SiC chip and DBC substrates by a x-ray observation. The results indicated that AlN-DBC can be as an optimization substrate for the best heat dissipation/durability in wide band-gap (WBG) power devices. Our results provide an important index for industries demanding higher power and temperature power electronics.

## 1. Introduction

Silicon carbide (SiC) and gallium nitride (GaN), are wide band-gap (WBG) semiconductors, are strongly recognized as the best materials for the power electronics applications demanding higher power and higher temperature [1,2]. Since these WBG semiconductors have superior properties, kind of a wide band gap (>3 eV), a high critical electric field (>3 MV/cm) and a high saturation velocity (>2 × 10^7^ cm/s), SiC and GaN can enable to overcome the ultimate performances reached by silicon (Si) based devices, in terms of power conversion efficiency [3]. In addition, WBG semiconductor devices can be operating much higher temperatures (>250 °C) than Si based devices (<150 °C) [4,5,6,7,8], this means massive, complex and heavy cooling systems can be eliminated from power conversion systems. Inverters and converters automotive components can be change to smaller by the simply heat dissipation design of in the high temperature environments [9]. In general, the structure of the power module has multi-layer structures of a semiconductor chip and a heat dissipation/insulation plate. All the layers have different material properties like a coefficient of thermal expansion (CTE), which causes thermos-mechanical stress during repetitive operating [10,11].

Besides, to withstand higher power and higher thermal density, how to insulate electricity and dissipate heat is one of the key issues of WBG power conversion systems. Therefore, there have been some issues, for instance how to interconnect these multi-layers to implement in high temperature and have a thermal-stable reliability. In addition, power electronic substrate plays an important role in dissipating heat to prevent power electronic module failure [12,13,14,15,16,17,18]. Heat dissipation/insulation substrate, which is a direct bonded copper (DBC) and a direct bonded aluminum (DBA), are existed between power semiconductor device and heat sink. Heat is transferred from surface of the power semiconductor device to the heat sink. The DBC and DBA were metallized both side of ceramic plate, to improve the thermal conductivity of ceramic substrates and form circuits [19], and were considered as the most promising substrates for power modules due to their excellent thermal conductivity, low thermal resistance, and high insulation voltage.

However, the sandwich structure of the DBC and DBA substrate will cause thermo-mechanical stresses due to the CTE mismatch. It has been reported that heat resistance of the substrate depending on the type of ceramics and metals [20,21,22]. In this regard, our group carried out to evaluate thermo-mechanical stability of various ceramic applied active metal brazed (AMB) DBC substrates with the Ni plating layer during a harsh thermal shock cycling test at the temperature range of from −50 °C to 250 °C [23]. The DBC substrate with silicon nitride (Si_3_N_4_-DBC) ceramics middle layer indicate no serious damage within 1,000 cycles of thermal shock cycles, while those of an aluminum nitride (AlN) and alumina (Al_2_O_3_) caused by Cu layer severe delamination after the same cycles [23].

**Figure 1:**
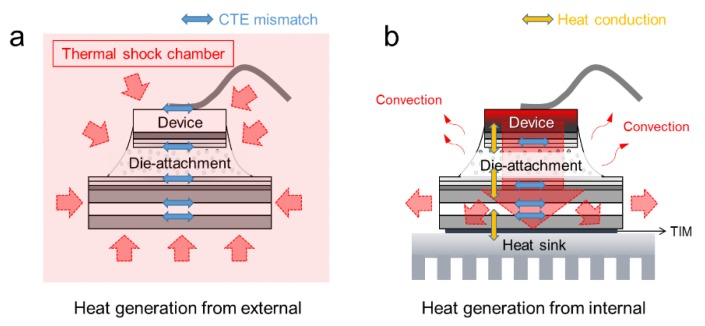
Comparison of the test environments and its heat transfer path: (**a**) thermal shock or aging condition as non-driving condition and (**b**) actual power cycling test as driving condition.

The results indicated that the failures may impress that the AlN and Al_2_O_3_ have critical disadvantage of higher CTE value and lower toughness than Si_3_N_4_. Since this previous study just investigated the DBC substrate itself, the failures must be induced by the mismatch between Cu and ceramic, meaning that the stress occurred from the DBC. Such thermal cycles give only homogeneous temperature change in the substrate specimens as shown in Figure 1a. However, the realistic thermal distributions in a ceramic substrate must have a large temperature gradient to transfer the generated heat from surface of the device chips to the cooling system as shown in Figure 1b. The stress induced by the CTE mismatch during the thermal conduction from chips to cooling system was more complexed than single DBC substrate. Therefore, the evaluation of such a practical temperature distribution is urgently needed for designing power module. In this context, power cycling test is the strongly useful evaluation methods to evaluate the reliability of a device packaging used in a condition similar to those in actual operations. In this process, the heat transfer performance of such a substrate mainly depends on the thermal properties of employed ceramic materials, e.g., Al_2_O_3_, AlN, and Si_3_N_4_. Power modules was required with a comprehensive combination of conductive Cu and insulation ceramics, involving the layer thicknesses and circuit patterns to achieve both thermal management and the least power loss at the same time. Usually, to measure the thermal resistance of devices after a power cycling test, it needs an equipment of power cycling system and also a T3ster system which means the Thermal Transient Tester system. It is a technology to monitor the thermal transport through the devices. However, both of the power cycling system and the T3ster system are very expensive and need to take up a lot of space. A simple, fast and miniaturized thermal measurement system is actually necessary.

In this study, SiC micro-heater chip was designed and used to bond with the various type of ceramic DBC substrates such as AlN and Al_2_O_3,_ and Si_3_N_4_. The Ag sinter joining was used as a die to attach material because which can withstand the high temperature applications and reported in many previous studies. Steady-state thermal resistance from the SiC micro-heater chip to cooling system with different type DBC were measured. In addition, the power cycling test also was performed to investigated the high temperature reliability of the SiC micro-heater chip die attach structure on each type DBC substrate. The failure after power cycling was analyzed by a micro-focus 3D computed tomography (CT) X-Ray system. This method can significantly distinguish with traditional thermal cycling test because thermal properties of material can be considered during repetitive thermal environments. Our method of power cycling test with SiC heater chip may provide in important evaluation index of future packaging systems with WBG semiconductors targeted for high-temperature/power density power modules.

## 2. Materials and Methods

### 2.1. SiC Micro-Heater Chip and Direct Bonded Copper (DBC) Substrate

**Figure 2:**
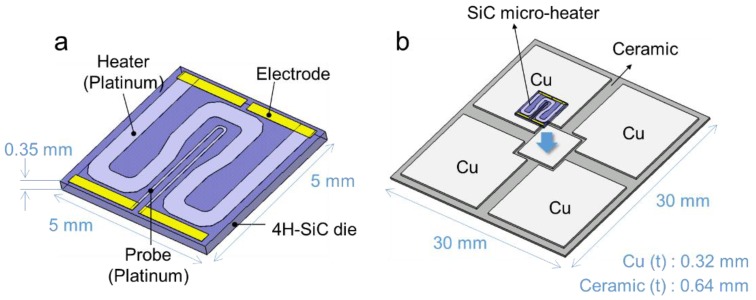
SiC heater die-attached structure: (**a**) SiC micro-heater chip structure (**b**) SiC heater chip on the substrate.

Geometrically 3D designed SiC micro-heater chip was fabricated with n-doped 4H-SiC by using a lithography technology (see Figure 2a), also it consists of a heater, a probe and an electrode, the material of heater and probe are platinum (Pt). The DBC substrate with five lands of the top side was introduced, and the SiC heater chip was die attached onto the center land and its geometric dimension is as displayed in Figure 2b. At the backside of SiC micro-heater chip and the top side of DBC substrates were metallized in the order of Ti and Ag layer by a sputtering process.

### 2.2. Die Attach Material

**Figure 3:**
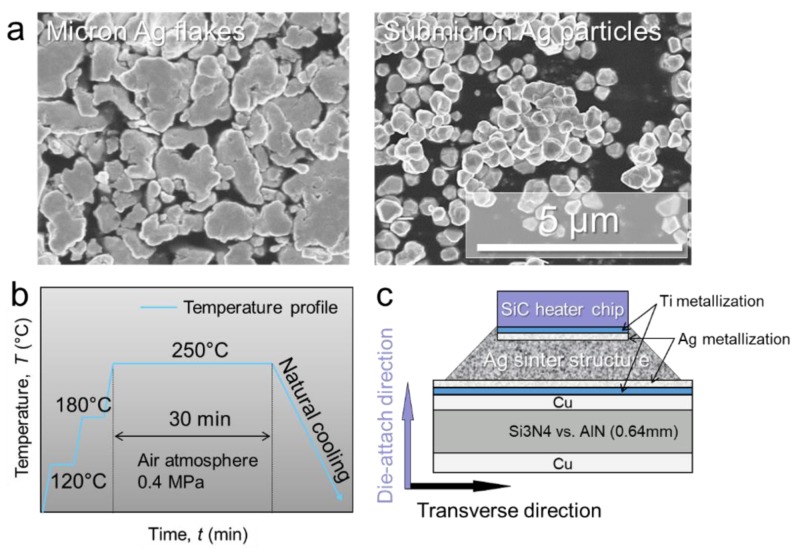
(**a**) Ag particles, (**b**) sintering condition and (**c**) schematic diagram of the assembled image.

Two types of Ag particles were mixed and used to make Ag paste. The first type comprised flake-shaped Ag particles with an average lateral diameter of 8 μm and a thickness of 260 nm, as shown in Figure 3a (AgC-239, Fukuda Metal Foil and Powder Co., Ltd., Kyoto, Japan). The second type comprised spherical-shape particles with an average diameter of 400 nm, as shown in Figure 3b (FHD, MitsuiMining and Smelting Co., Ltd., Tokyo, Japan). The two kinds of Ag particles were mixed as the Ag filler with a weight ratio of 1:1 [24]. These hybrid particles were then stirred magnetically for 10 min and vibrated ultrasonically for 30 min with viscosity of 100–200 Pa·s solvent (alkylene glycols and polyols) using a hybrid mixer (HM-500, Keyence Corporation, Osaka, Japan) to fabricate Ag paste to achieve a uniform mixture. The amount of the solvent was maintained at approximately 10 wt % in the paste to keep a suitable viscosity of 150–250 centi poise (cPs) at room temperature. The Ag paste was then printed onto the center land of the DBC substrates which were purchased from Mitsubishi Materials Corporation. For the bonding process, a step-by-step profile with low pressure (0.4 MPa), including 120 °C and 180 °C for 5 min as pre-heating, and then 250 °C for 60 min, was applied to prevent pores formation caused by evaporation of organic solvent (see Figure 3b). Finally, the sintered cross section is formed as shown in Figure 3c. For power supply, 10EA Au wires of 45 μm diameter (TANAKA DENSHI KOGYO, Yoshida, Japan) on the four electrode were each interconnected by an ultrasonic wire bonding machine (Kulicke & Soffa Industries Inc, 4500 digital series, Singapore).

### 2.3. Test Machine and Method

**Figure 4:**
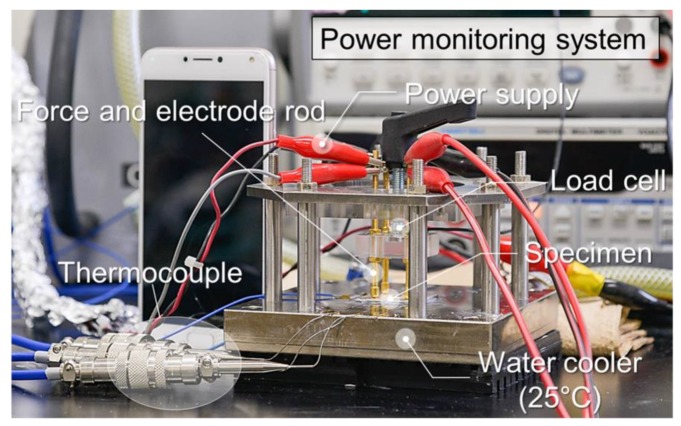
Power cycling test system with SiC micro-heater chip.

Figure 4 shows the machine of power cycling test and thermal resistance measurement and its component. The machine consists of four parts, power supplier, controller, monitor and water-cooling system. Over 200 W of power is supplied from the power source to the electrode, and when heat is generated from the top of the heater chip, heat is transferred to the heat sink (25 °C) through the die attach and the DBC substrate. At this time, SiC heater/DBC die-attached specimen is mounted on the water cooling plate by diamond thermal grease (thermal interface material, TIM). To prevent delamination between specimen and cooling plate during power cycling, the DBC substrate was fixed to the water-cooling plate with a force of 20 N. Then, Δ*T* is calculated by the temperature of the top surface of the SiC heater chip (*T_chip_*) and the temperature of the thermocouple attached to the water-cooling plate (*T_thermocouple_*), the equation is as follows:

(1) $$\Delta T=T_{chip}-T_{thermocouple}$$

Here, the thermal resistance (*R_th_*) can be calculated by dividing this temperature difference (Δ*T*) by the input power (*Q*) as follows:

(2) $$R_{th}=\Delta T/Q$$

where, *Q* (W) is Joule heating by the electrical power of SiC micro-heater chip and *T* is the temperature (K).

## 3. Results and Discussion

### 3.1. Steady-State Thermal Resistance

**Figure 5:**
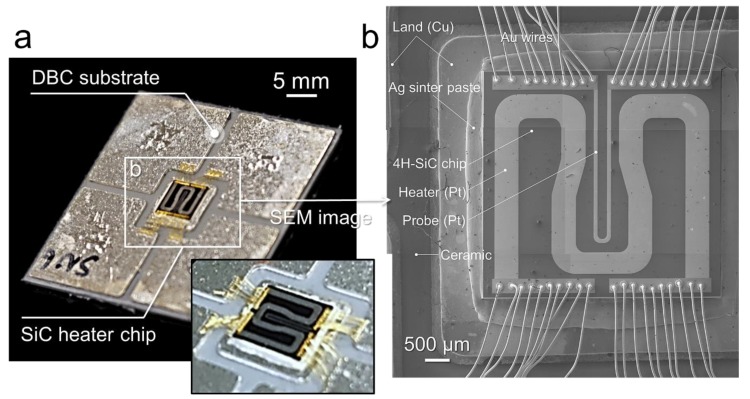
Test specimen description: (**a**) interconnected SiC heater chip on direct bonded copper (DBC) substrate and (**b**) its SEM image of detail components.

**Figure 6:**
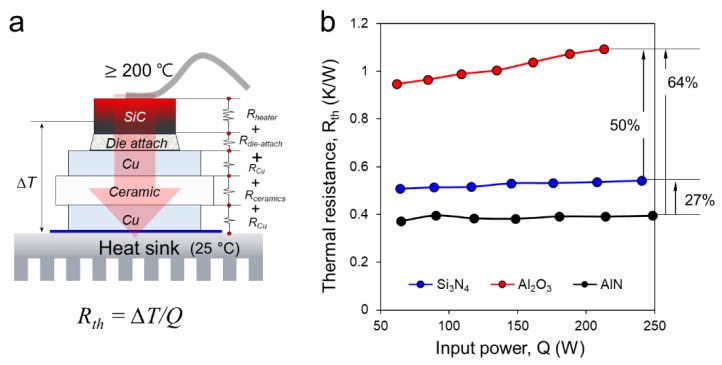
(**a**) Schematic diagram of heat path and its thermal resistance measurement during power cycling (**b**) results of various ceramics of thermal resistance.

For thermal resistances measurement, a specimen was packaged by the method introduced in the previous section (see Figure 3). After the die bonding, the specimen interconnected without noticeable defects such as oxidation, and its actual manufactured and interconnected SiC heater die attached specimen are shown in Figure 5a. Furthermore, the electrode and temperature sensor of SiC heater chip was Au wire bonded with the DBC substrate after die-attach bonding. The Au wire bonded with the DBC substrate was given at upper left corner in Figure 5a. In addition, an enlarged image of the packaged SiC micro-heater chip circuit is shown in Figure 5b. When the power is supplied, the heat transfer path is shown in Figure 6a, where each layer has a thermal resistance. Here, since all materials except ceramic are the same, input power dependent thermal resistance performance can be directly compared according to ceramic type. The results of thermal resistance performance are shown in Figure 6b. The thermal resistance of the SiC micro-heater chip power modules on the DBC-Al_2_O_3_ substrate at power over 200 W was about twice higher than DBC-Si_3_N_4_ and also higher than DBC-AlN. The thermal resistance of DBC-AlN was lower than that of DBC-Si_3_N_4_. In addition, thermal resistance of the SiC micro-heater chip power modules on the DBC-Al_2_O_3_ substrate tended to increase thermal resistance significantly with increasing power but there is almost not so much change for the DBC-AlN and DBC-Si_3_N_4_ with the increased power. The reason of the lowest thermal resistance of AlN is that the thermal conductivity of AlN (285 W·m^−1^·K^−1^, for single crystals) is significantly higher than Al_2_O_3_ (25 W·m^−1^·K^−1^) and Si_3_N_4_ (60–90 W·m^−1^·K^−1^) [21,25]. Therefore, AlN-DBC can be considered to have the best heat dissipation performance of an excellent power module when applied properly in the area where heat dissipation is intensively required.

### 3.2. Power Cycling Test

**Figure 7:**
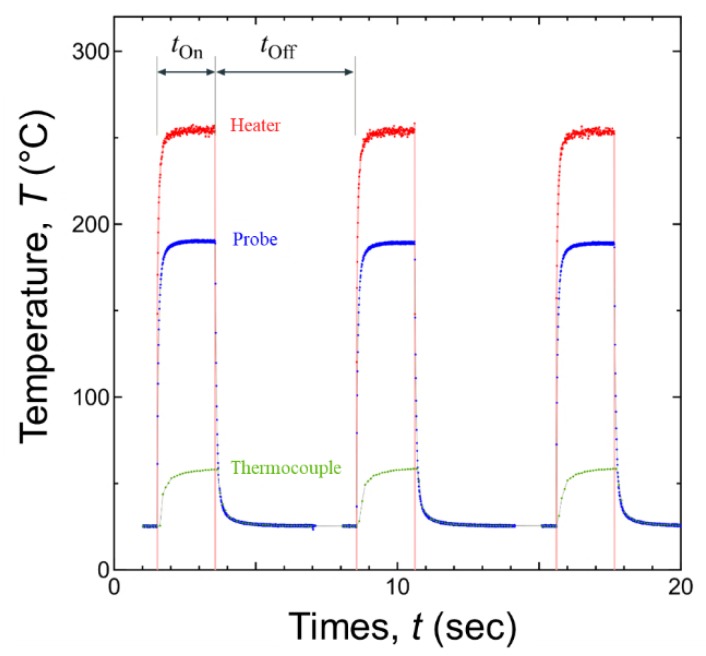
Temperature swing during power cycling tests.

For the power cycling test, the temperature of the cooling plate was constantly controlled to be 50 °C. The water flow rate was 5.4 L/min. The constant current for heating were tested, and constant current is necessary to suppress the surge spike when heating was started. The typical temperature profile of the power cycling is plotted in Figure 7. The input electric current in the test was 2.1 A. The condition of power cycling test was applied 2 s ON and 5 s OFF at the input power of 200 W.

**Figure 8:**
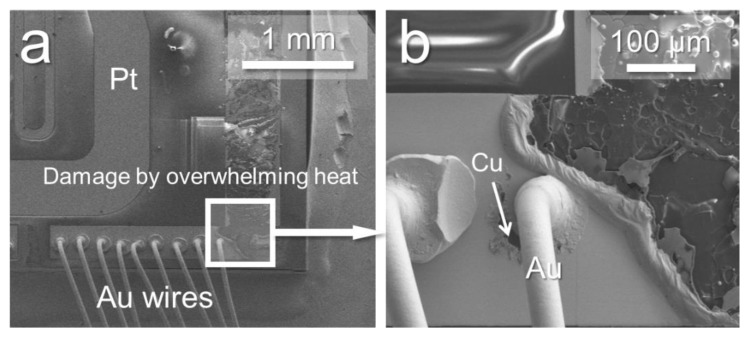
Pt heater pattern line was partly broken (**a**) and its magnified view (**b**).

The results indicated that Al_2_O_3_ was stopped during the power cycle test due to excessive rise in thermal resistance before 1000 cycles, Si_3_N_4_ and AlN specimens were each subjected to more than 20,000 cycles of power cycle tests. In the case of Al_2_O_3_ stopping, die-attach and ceramic component were not any degradation but the Pt heater pattern line was partially broken as shown in Figure 8a due to the poor heat dissipation performance of Al_2_O_3_. The Pt heater pattern line accumulated a lot of heat, leading to the temperature increase significantly, leading to the film of pattern line partially melted as shown in Figure 8b.

**Figure 9:**
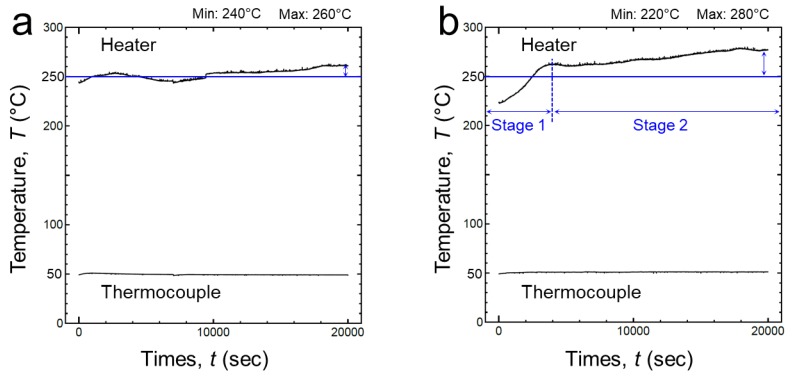
Temperature swing behavior during power cycling tests: (**a**) Si_3_N_4_ and (**b**) AlN.

Figure 9a shows temperature swing behavior of the Si_3_N_4_-DBC substrate, the heater temperature at the beginning was about 240 °C, and when it reached 20,000 cycles, the temperature was about 260 °C. In the case of AlN-DBC substrate, the starting temperature was about 220 °C, and the temperature after 20,000 cycles was about 280 °C (see Figure 9b). The temperature ranges of Si_3_N_4_ and AlN are not significantly different, but the temperature change tendency was remarkable. Unlike Si_3_N_4_ substrate, which has a small change in temperature, AlN was largely divided into two stages. The stage 1 is from the starting point to 4000 cycles, it reached up to saturation temperature. The stage 2 is from 4000 cycles to 20,000 cycles, at a relatively stable temperature, the temperature slowly reached its peak point.

**Figure 10:**
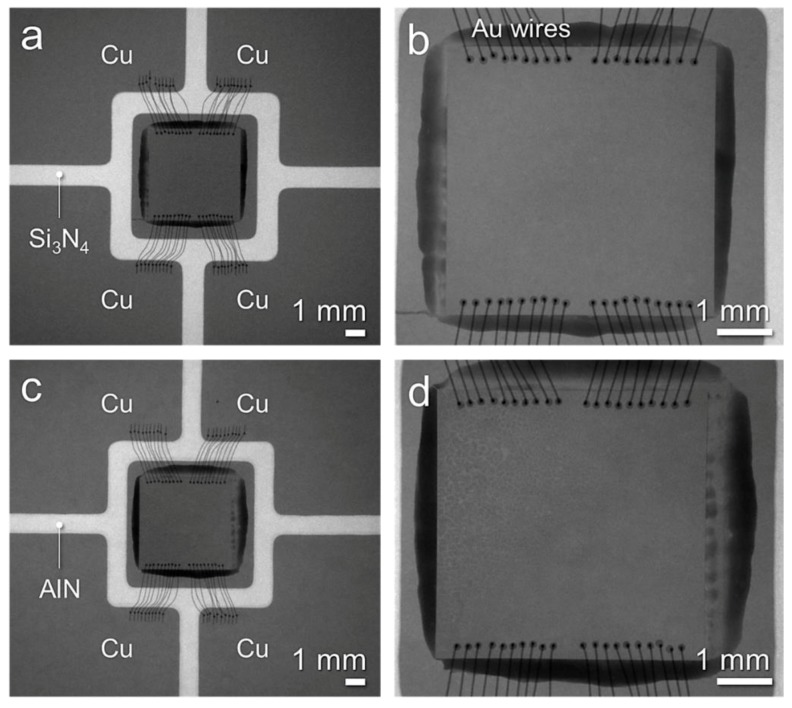
Nondestructive test results (X-ray) after power cycling tests (20,000 cycles): (**a**) specimen of Si_3_N_4_ and (**b**) its high magnification image, (**c**) specimen of AlN and (**d**) its high magnification image.

**Figure 11:**
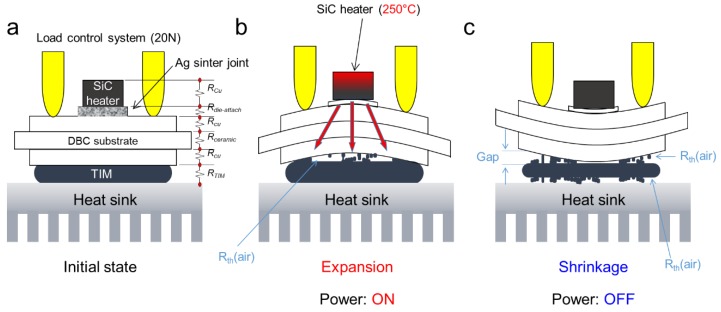
Schematic diagram of AlN temperature rise mechanism by mechanical behavior during power cycling test: (**a**) initial state (**b**) expansion (**c**) shrinkage.

To investigate a factor of different temperature behavior, a nondestructive test was performed by a micro-focus 3D computed tomography (CT) X-Ray system (XVA-160N, Uni-Hite System Corporation, Yamato City, Japan) on the specimens after power cycling tests. Figure 10 displays nondestructive test results (X-ray) after power cycling tests after power cycling tests (20,000 cycles). Figure 10a,b show the SiC micro-heater chip power modules on the DBC-Si_3_N_4_ substrate and its high magnification image, respectively. Figure 10b,c the SiC micro-heater chip power modules on the DBC-Si_3_N_4_ substrate of AlN and its high magnification image Figure 10d, respectively. There was no relationship between the different temperature behavior of the two substrates and the substantial damage to the die attach structure. Therefore, no delamination of the ceramic or degradation of the Ag sinter joint occurred during power cycling tests, and it was clearly found that the temperature rise did not cause physical failure. Despite the excellent thermal conductivity of AlN, the cause of temperature rise shown in Figure 9b can be regarded as an issue of the TIM. The mechanism of air entrainment into the TIM are shown in Figure 11. In the initial state, the DBC substrate and the water cooling plate were closely adhered by TIM (see Figure 11a), after experiencing expansion and shrinkage, there should be some gaps generation between the TIM and substrate, the adhesion strength changed to lower with a little gap generation as displayed in Figure 11b,c. Air entrainment into the TIM also can lead to the temperature swing increase.

Thus, to avoid this phenomenon, it can be prevented by using a soft TIM or warpage suppression mechanism that does not harden under repeated substrate warpage behavior. The results of the power cycling test using the SiC micro-heater chip show the heat resistance performance of the DBC substrate, are significantly different from the traditional thermal shock test. Consequently, the AlN-DBC substrates may have the best heat dissipation and durability, perhaps by optimizing TIM performance for WBG power conversion systems.

## 4. Conclusions

In this study, a power cycling test system based on a SiC micro-heater chip was developed to simulate the active SiC devices where the temperature of the heater chip can be recorded through the power cycling test in real-time. The new approach is useful for the evaluation of high-power devices with a similar condition to the real packaging system. The thermal resistance of of the SiC micro-heater chip power modules on DBC-AlN was lower than that of DBC-Al_2_O_3_ and DBC-Si_3_N_4_. In addition, thermal resistance of the SiC micro-heater chip power modules on the DBC-Al_2_O_3_ substrate tended to increase thermal resistance significantly with increasing power but there is almost not so much change for the DBC-AlN and DBC-Si_3_N_4_ with the same increased power. Furthermore, no delamination of the ceramic or degradation of the Ag sinter joint occurred during power cycling tests for both DBC-AlN and DBC-Si_3_N_4_, and it was clearly found that the temperature rise did not cause physical failure. The results exhibit that both DBC-AlN and DBC-Si_3_N_4_ are excellent candidates for high power modules with WBG semiconductor devices, suitable for industries demanding high energy efficiency, when the packaging like the die-attach and the cooling system is well optimized.
